# Obesity and Ventilatory Responses During Exercise in the Fitness Registry and the Importance of Exercise National Database (FRIEND)

**DOI:** 10.1111/sms.70264

**Published:** 2026-03-19

**Authors:** Thomas G. Bissen, Ross Arena, Matthew P. Harber, Leonard A. Kaminsky, Jonathan Myers, Joseph C. Watso

**Affiliations:** ^1^ Cardiovascular and Applied Physiology Laboratory Florida State University Tallahassee FL USA; ^2^ Institute of Sports Sciences and Medicine Florida State University Tallahassee FL USA; ^3^ Healthy Living for Pandemic Event Protection (HL ‐ PIVOT) Network Chicago IL USA; ^4^ Department of Physical Therapy University of Illinois Chicago Chicago IL USA; ^5^ Health Sciences Taylor University Upland IN USA; ^6^ Fisher Institute of Health and Well‐Being Ball State University Muncie IN USA; ^7^ Division of Cardiovascular Medicine VA Palo Alto Health Care System CA USA; ^8^ Cardiovascular Institute Stanford University Stanford CA USA; ^9^ Institute for Successful Longevity Florida State University Tallahassee FL USA

**Keywords:** body mass index, cardiopulmonary exercise test, cardiorespiratory fitness, V̇_E_/V̇CO_2_ slope

## Abstract

A high minute ventilation/rate of carbon dioxide production (V̇_E_/V̇CO_2_) slope during exercise is prognostic for cardiovascular mortality among clinical populations. Obesity represents a major modifiable risk factor for cardiovascular disease. However, it is unclear whether body mass index (BMI) is associated with V̇_E_/V̇CO_2_ slope among apparently healthy adults. Therefore, we used the Fitness Registry and the Importance of Exercise National Database (FRIEND) to determine whether BMI is positively associated with V̇_E_/V̇CO_2_ slope in the context of apparently healthy adults. All participants completed a cardiopulmonary exercise test on a cycle ergometer. Linear regressions adjusted for age, sex, and race/ethnicity were used to compare the V̇_E_/V̇CO_2_ slope between adults with and without obesity (BMI </≥ 30 kg/m^2^). Partial correlation adjusted for age, sex, race/ethnicity, and cardiorespiratory fitness was used to determine the relation between the V̇_E_/V̇CO_2_ slope and BMI. All data are presented as median [IQR]. We set *α* a priori to < 0.05. The sample (*n* = 3534) characteristics were as follows: (1) age = 40 (17) years; (2) 20% female; (3) cardiorespiratory fitness = 27.8[10.8] mL O_2_●kg^−1^●min^−1^ & 2.3[0.9] L O_2_●min^−1^; and (4) BMI = 26.1[5.0] kg/m^2^. V̇_E_/V̇CO_2_ slope was higher in adults with obesity 25.0[3.5] compared to those without obesity 24.7[3.6] with a negligible effect size (*R* ^2^ = 0.132, adjusted *R* ^2^ = 0.131, F_4,3529_ = 134, *p* < 0.001). V̇_E_/V̇CO_2_ slope was weakly associated with BMI across the cohort (ρ = 0.079, *p* < 0.001). Obesity was positively, but negligibly, associated with a higher V̇_E_/V̇CO_2_ slope in the FRIEND Registry.

**Abbreviations**
BMI: body mass index
CPET: cardiopulmonary exercise test
FRIEND: fitness registry and the importance of exercise national database
NRL: neutrophil‐to‐lymphocyte ratio
V̇CO_2_
: rate of carbon dioxide production
V̇_E_
: minute ventilation
V̇_E_/V̇CO_2_ slope: minute ventilation/rate of carbon dioxide production
V̇O_2_
: rate of oxygen consumption

## 1 Introduction

Cardiopulmonary exercise testing (CPET) detects myocardial ischemia [1, 2], predicts the progression of cardiorespiratory diseases [3, 4], and determines exercise capacity (i.e., peak oxygen consumption (V̇O_2peak_)) [5, 6, 7]. Moreover, higher cardiorespiratory fitness (CRF) is strongly associated with lower all‐cause mortality [6, 8, 9]. The generation of large databases has allowed for the development of normative data for individual responses during CPET [10, 11]. CPET provides the rate of carbon dioxide production (V̇CO_2_) and the compensatory increase in pulmonary minute ventilation (V̇_E_), known as the V̇_E_/V̇CO_2_ slope, often used to determine ventilatory responses with the context of metabolic demand. Additionally, CPET provides CRF via V̇O_2peak_. The V̇_E_/V̇CO_2_ slope is prognostic for all‐cause mortality in clinical populations (e.g., cardiac patients) [6, 8, 9, 12, 13]. Furthermore, the V̇_E_/V̇CO_2_ slope is a stronger predictor of cardiac‐related mortality compared to exercise capacity (i.e., V̇O_2peak_) [14, 15, 16, 17].

An elevated V̇_E_/V̇CO_2_ slope is present in various disease states, in part representing a ventilation‐perfusion mismatch indicative of ventilatory inefficiency [18]. This has been observed in patients with heart failure who exhibit excess V̇_E_, resulting in an increased V̇_E_/V̇CO_2_ slope during exercise [19]. Ventilatory inefficiency is linked to a reduced ability to perform activities of daily living [20]. Furthermore, among adults with heart failure with preserved ejection fraction, the rate of obesity is greater than 80% [21]. In addition, obesity is one of four modifiable risk factors that account for 62% of the total risk of heart failure [22]. Specifically, every 1 unit higher body mass index (BMI) results in a ~6% increase in risk for developing heart failure [23]. A clearer understanding of how obesity, in isolation, influences ventilatory efficiency is warranted.

Obesity has become a global epidemic; approximately 890 million adults today have obesity, which costs nearly $2 trillion globally in 2020 [24]. Adults with obesity experience mechanical ventilatory constraints associated with excess adiposity [25]. Additionally, adults with obesity develop reduced functional residual capacity and expiratory reserve volume, leading to breathing near residual volumes [26, 27]. The normal healthy range for the V̇_E_/V̇CO_2_ slopes from rest to peak exercise are in the mid‐20s, with values below 30 considered normal [28]. That said, there has been discussion on whether to calculate V̇_E_/V̇CO_2_ slopes up to the ventilatory threshold or to calculate the full slope to peak exercise. It has been suggested that using the entire test (i.e., peak) to calculate is optimal for prognostic sensitivity [29]. Furthermore, clinical populations of adults with obesity (defined as BMI ≥ 30 kg/m^2^) have augmented V̇_E_/V̇CO_2_ slopes. Originally thought to be BMI‐independent [30], patients with coronary artery disease [31] and heart failure [32] with overweight or obesity exhibit high V̇_E_/V̇CO_2_ slopes.

Some studies suggest no relation between BMI and the V̇_E_/V̇CO_2_ slope [30, 33] while other reports suggest that a higher BMI is associated with a lower V̇_E_/V̇CO_2_ slope among adults with BMI values ranging from 30 to 80 kg/m^2^ [34, 35]. Additionally, across this large BMI range, males demonstrate a larger blunting effect with lower V̇_E_/V̇CO_2_ slopes for each BMI subgroup [35]. This blunted V̇_E_/V̇CO_2_ slope response to exercise indicates inadequate increases in ventilation during exercise, suggesting a failure to increase V̇_E_ proportional to the metabolic demands of exercise. In summary, there is conflicting information on the association between BMI and the V̇_E_/V̇CO_2_ slope.

The distinction between adults with and without obesity has shown that adults with obesity have altered responses during CPET; however, subgroup analysis of BMI (normal weight, overweight, and obese) also warrants investigation. The previous studies that observed the association between BMI and the V̇_E_/V̇CO_2_ slope were of small to modest sample sizes [35, 36, 37]. Additionally, previous studies included participants with obesity but did not isolate adults with disease‐free obesity effectively [37]. Therefore, we used the Fitness Registry and the Importance of Exercise National Database (FRIEND) to test the hypothesis that adults with obesity would have an augmented V̇_E_/V̇CO_2_ slope compared to adults without obesity in the context of apparently healthy adults.

The FRIEND database has two published works that addressed V̇_E_/V̇CO_2_ slope reference values, but both focused on treadmill tests and did not provide a substantial investigation of the role of obesity [38, 39]. There has been one additional study focused on the V̇_E_/V̇CO_2_ slope and hypertension that specifically excluded obesity [40]. Thus, the present analysis examining the association between BMI subgroups and V̇_E_/V̇CO_2_ slope during upright cycling exercise is a warranted and novel investigation.

## 2 Methods

### 2.1 Database

The Fitness Registry and the Importance of Exercise National Database (FRIEND) was created in 2014 at the Clinical Exercise Physiology Program at Ball State University. The FRIEND Registry is a large database (> 126 000 CPETs) from multiple laboratories worldwide that have been used to establish normative values for physiological responses to exercise across the lifespan [41]. Laboratories contributing to the FRIEND registry completed all tests consistent with exercise testing recommendations [42, 43, 44]. Additionally, all data from laboratories and centers that contributed data to the FRIEND Registry had to pass quality assurance from FRIEND prior to entry. Approval for use of the de‐identified database for research was obtained from the Institutional Review Board at Ball State University.

### 2.2 Participants

Participant demographics included male/female, self‐reported age, race, ethnicity, the presence of chronic disease, current medication usage, and smoking status. For this analysis, we excluded those with current smoking status, people < 18 years of age, those with a pathological exercise test indication (e.g., excessive dyspnea upon exertion), any medication usage, and any known chronic diseases or conditions. Specifically, participants with self‐reported coronary artery disease, hypertension, stroke, peripheral artery disease, heart failure, any cardiomyopathies, valvular disease, hyperlipidemia, endocrine conditions, cancer, chronic kidney disease, diabetes, liver disease, neurological disease, asthma, chronic obstructive pulmonary disease, and any pulmonary restrictive diseases (e.g., chronic bronchitis) were excluded from the current analysis. Resting blood pressure was measured before the CPET in the seated position. Additionally, BMI was calculated before testing using body height and mass.

### 2.3 Exercise Testing

Given heterogeneous responses to different exercise modalities and the FRIEND database having a higher number of upright cycle ergometer tests, we exclusively examined upright cycle ergometer CPETs. Additionally, cycle ergometers increase only resistance (wattage) while treadmill tests increase grade and velocity. In this context, adjustments in a single exercise intensity variable reduced unnecessary variability in exercise responses that are not a result of obesity. Therefore, this dataset includes only CPETs performed on an upright cycle ergometer (weight‐independent exercise). Continuous monitoring of V̇_E_, V̇CO_2_, and V̇O_2_ via expired gases was used to calculate V̇_E_/V̇CO_2_ slope and V̇O_2peak_. All included CPETs had peak respiratory exchange ratios > 1.00. Peak data was determined from the last 30 or 60 s of each test. In accordance with previous studies [40, 45], the V̇_E_/V̇CO_2_ slope was calculated from the onset of exercise to peak exercise via least‐squares linear regression (y = mx + b; “m” = slope). Calculating the slope across the entire test may improve prognostic sensitivity [29].

### 2.4 Data & Statistical Analysis

There is no consensus on the minimum important clinical difference in V̇_E_/V̇CO_2_ slope. Thus, we did not conduct an a priori sample size estimation. Variables were tested for normality (Shapiro–Wilk test of normality, *p* < 0.05). Nonparametric testing was used for all analyses with at least one non‐normally distributed variable. Thus, we presented non‐normal data as the median [interquartile range].

Centers for Disease Control and Prevention criterion for obesity in adults (≥ 30.0 kg/m^2^) was used. Group characteristic comparisons were performed using Mann–Whitney U tests (rank biserial correlation was the effect size estimate) and *X* ^2^ tests (Cramer's V was the effect size). In addition to the dichotomous grouping, participants were split into three groups according to BMI: normal weight (18.5–24.9 kg/m^2^), overweight (25.0–29.9 kg/m^2^), and obesity (≥ 30.0 kg/m^2^). One‐way ANOVA, or the Kruskal–Wallis test, was used to determine differences in subgroup characteristics. Finally, the proportion of participants with a V̇_E_/V̇CO_2_ slope > 45 between groups was determined because it has been used as a clinical cut‐off for higher mortality risk [46].

The primary hypothesis was assessed by comparing the V̇_E_/V̇CO_2_ slope between adults with obesity and those without obesity using linear regression. Subgroups were then compared, including those with normal weight, overweight, and obesity using linear regression. These analyses were repeated after covariate adjustment with absolute V̇O_2peak_, age, sex, and ethnicity based on previous literature suggesting these variables independently influence the V̇_E_/V̇CO_2_ slope [38, 39, 47, 48]. Next, Spearman's rank partial correlations were used to assess the association between BMI and the V̇_E_/V̇CO_2_ slope with and without covariate adjustments.

The current analysis calculated effect sizes to aid interpretation in addition to *p*‐values. Rank–biserial correlation effect sizes were defined as small (0–0.19), medium (0.20–0.30), large (0.30–0.39), and very large (0.40–1). Eta squared values were defined as small (0.01), medium (0.06), and large (0.14). Cramer's *V* effect sizes were defined as small, medium, and large based on the degrees of freedom and Cramer's *V* value [49, 50]. For linear regression tests, *R* ^2^ was used for effect sizes, with 0.3 as small, 0.5 as medium, and 0.7 as a large effect [51]. For partial correlations, Spearman's rho was used for effect size with 0.10 as small, 0.30 as medium, and 0.50 as a large effect size [52]. The absence of collinearity was confirmed by excluding covariates with VIF > 2.5. Data analysis was conducted using Jamovi (version 2.6.44) and GraphPad Prism (version 10.5.0 for Windows, GraphPad Software, San Diego, CA, USA). We set *α* a priori to 0.05.

## 3 Results

### 3.1 Participants

The characteristics of the entire sample (*n* = 3534) were age: 40 [17] years; 20% female; V̇O_2peak_: 27.8 [10.8] mL O_2_●kg^−1^●min^−1^ and 2.3 [0.9] L O_2_●min^−1^; and BMI 26.1 [5.0] kg●m^−2^. Included in the analysis are tests from 1992 to 2021. The testing locations were as follows: < 1% (*n* = 11) unknown, < 1% (*n* = 1) from Antigua and Barbuda, 1% (*n* = 35) from the United States Virgin Islands, 4% (*n* = 127) from Switzerland, 5% (*n* = 193) from Canada, and 90% (*n* = 3167) from the United States.

### 3.2 No Obesity Versus Obesity

We present group characteristics for adults with and without obesity in Table 1. Age, male: female ratio, race/ethnicity, resting blood pressure, peak workload, and absolute CRF differed between groups, but the effect sizes were small. By design, adults with obesity had a higher (*p* < 0.001) BMI than adults without obesity. Additionally, adults with obesity had a higher (*p* < 0.001) body mass and lower (*p* < 0.001) physical fitness compared to adults without obesity, with very large effect sizes (Table 1).

**TABLE 1:** Participant characteristics among adults without and with obesity.

	Without obesity (*n* = 2927)	With obesity (*n* = 607)	*p*	Effect size
Age (years)	40 (18)	42 (15)	< 0.001	0.09 (small)
Male/Female (%)	80 M; 20F	85 M; 15 F	< 0.001	0.047 (small)
Race/Ethnicity (%)	5 UK, < 1 AI, 3 A, 13 B, 7 H, 3 O, 68 W	2 UK, < 1 AI, 1 A, 21 B, 11 H, 4 O, 60 W	< 0.001	0.129 (small)
Body mass (kg)	78.6 (17.0)	101.2 (13.4)	< 0.001	0.83 (very large)
Body mass index (kg/m^2^)	25.2 (4.1)	32.0 (2.8)	< 0.001	1.00 (very large)
Resting systolic blood pressure (mmHg)	110 (14)	114 (11)	< 0.001	0.17 (small)
Resting diastolic blood pressure (mmHg)	70 (8)	72 (6)	< 0.001	0.17 (small)
Peak workload (watts)	200 (75)	200 (76)	0.04	0.02 (small)
Absolute V̇O_2_ (peak L O_2_/min)	2.28 (0.95)	2.40 (0.91)	0.009	0.07 (small)
Relative V̇O_2_ (peak mL O_2_/kg/min)	28.9 (10.7)	23.0 (8.2)	< 0.001	0.44 (very large)

*Note:* We present values as Median (IQR) or %. We compared group medians using Mann Whitney *U* tests (with rank biserial correlation as the effect size estimate). We compared proportions using X^2^ tests (with Cramer's V as the effect size estimate).

Abbreviations: A, asian; AI, American Indian; B, black; F, female; H, hispanic; M, male, O, other; Race/ethnicity: UK, unknown or unreported; W, white.

**FIGURE 1:**
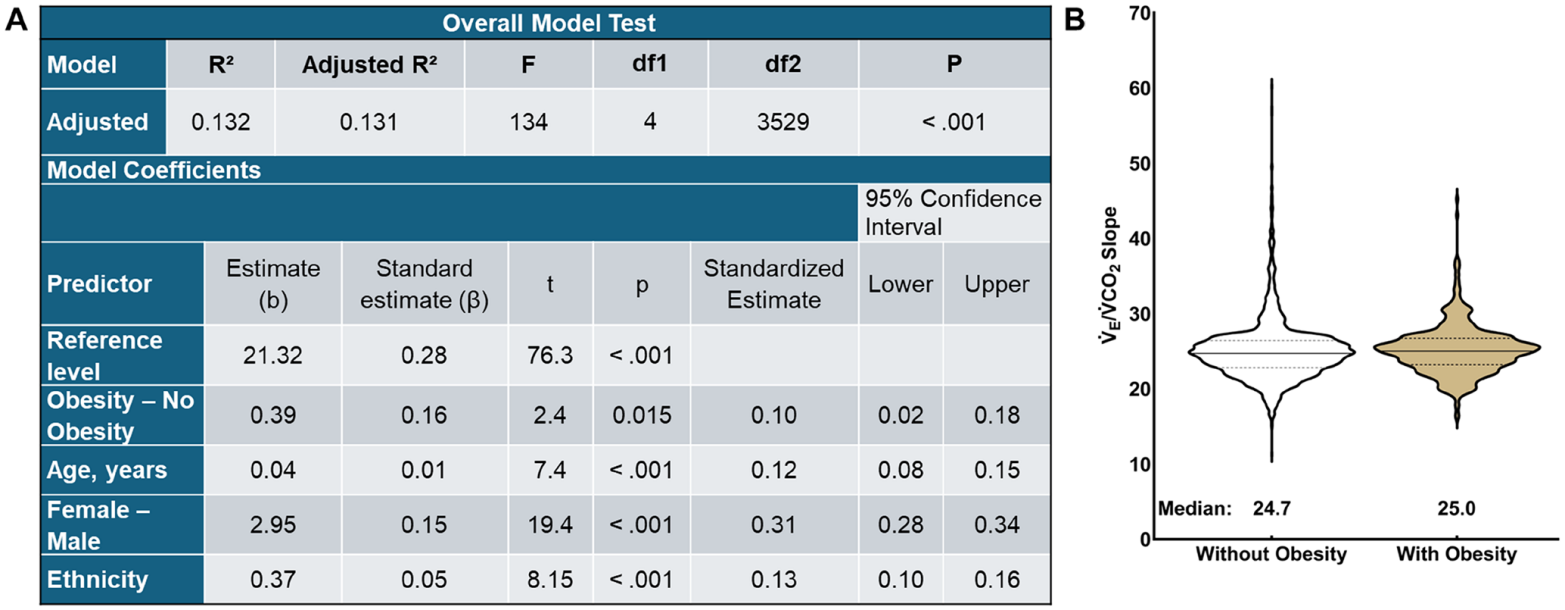
(A) Linear regression for V̇_E_/V̇CO_2_ slope in adults with and without obesity adjusted for age, sex, and race/ethnicity. (B) Violin plot of V̇_E_/V̇CO_2_ slope in adults without and with obesity. V̇_E_/V̇CO_2_ slope = minute ventilation/rate of carbon dioxide production.

In an unadjusted analysis, V̇_E_/V̇CO_2_ slope did not differ between adults with and without obesity (*R* ^2^ < 0.001, adjusted *R* ^2^ < 0.001, F_1,3532_ = 1.35, *p* = 0.245). Absolute V̇O_2peak_ was removed from the model because it was not a significant covariate (*p* = 0.899). In the covariate adjusted analysis, the V̇_E_/V̇CO_2_ slope was greater (*p* < 0.001) in adults with obesity compared to those without obesity (Figure 1), with a negligible effect size.

### 3.3 Subgroup Analyses

Subgroup characteristics for adults with normal weight, overweight, and obesity are presented in Table 2. Age, resting blood pressure, and peak workload differed between groups, but the effect sizes were small. By design, BMI differed between subgroups in a stepwise manner. There were large effect sizes for differences in the male:female ratio and race/ethnicity proportions between groups. Body mass differed between subgroups in a stepwise manner with a large effect size. Adults in the normal weight group had lower (*p* < 0.001) absolute but higher (*p* < 0.001) relative CRF relative to the other subgroups, with medium effect sizes (Table 2).

**TABLE 2:** Participant characteristics among adults in BMI subgroups.

	Normal weight (*n* = 1338)	Overweight (*n* = 1589)	Obesity (*n* = 607)	*p*	Effect size
Age (years)	39 (20)^a^, ^b^	40 (16)	42 (15)	< 0.001	0.009 (small)
Male/Female (%)	70 M; 30 F^a^, ^b^	88 M, 12 F	85 M, 15 F	< 0.001	0.211 (large)
Race/Ethnicity (%)	8 UK, < 1 AI, 5 A, 12 B, 5 H, 3 O, 66 W^a^, ^b^	1 UK, < 1 AI, 2 A, 14 B, 9 H, 3 O, 70 W^b^	2 UK, < 1 AI, 1 A, 21 B, 11 H, 4 O, 60 W	< 0.001	0.163 (large)
Body mass (kg)	70.2 (13.9)^a^, ^b^	85.6 (11.9)^b^	101.0 (13.7)	< 0.001	0.579 (large)
Body mass index (kg/m^2^)	23.0 (2.5)^a^, ^b^	27.1 (2.3)^b^	32.0 (2.8)	< 0.001	0.850 (large)
Resting systolic blood pressure (mmHg)	110 (16)^a^, ^b^	112 (14)^b^	114 (11)	< 0.001	0.021 (small)
Resting diastolic blood pressure (mmHg)	70 (9)^a^, ^b^	70 (8)^b^	72 (6)	< 0.001	0.021 (small)
Peak workload (watts)	185 (80)^a^, ^b^	205 (75)^b^	200 (76)	< 0.001	0.027 (small)
Absolute V̇O_2_ (peak L O_2_/min)	2.11 (0.89)^a^, ^b^	2.42 (0.89)	2.40 (0.91)	< 0.001	0.040 (medium)
Relative V̇O_2_ (peak mL O_2_/kg/min)	30.0 (11.6)^a^, ^b^	28.1 (10.1)^b^	23.0 (8.2)	< 0.001	0.096 (medium)

*Note:* We present values as Median (IQR) or %. We compared group medians using Kruskal–Wallis tests (ε) as the effect size estimate. We compared proportions using *X* ^2^ tests (with Cramer's *V* as the effect size estimate).

Abbreviations: A, asian; AI, american Indian; B, black; F, female; H, hispanic; M, male, O, other; Race/ethnicity: UK, unknown or unreported; W, white.

^a^ Post hoc analyses denote *p* < 0.05 using Dwass–Steel–Critchlow–Fligner post hoc pairwise comparisons vs. overweight^2^ or obesity.

^b^ Obesity group.

**FIGURE 2:**
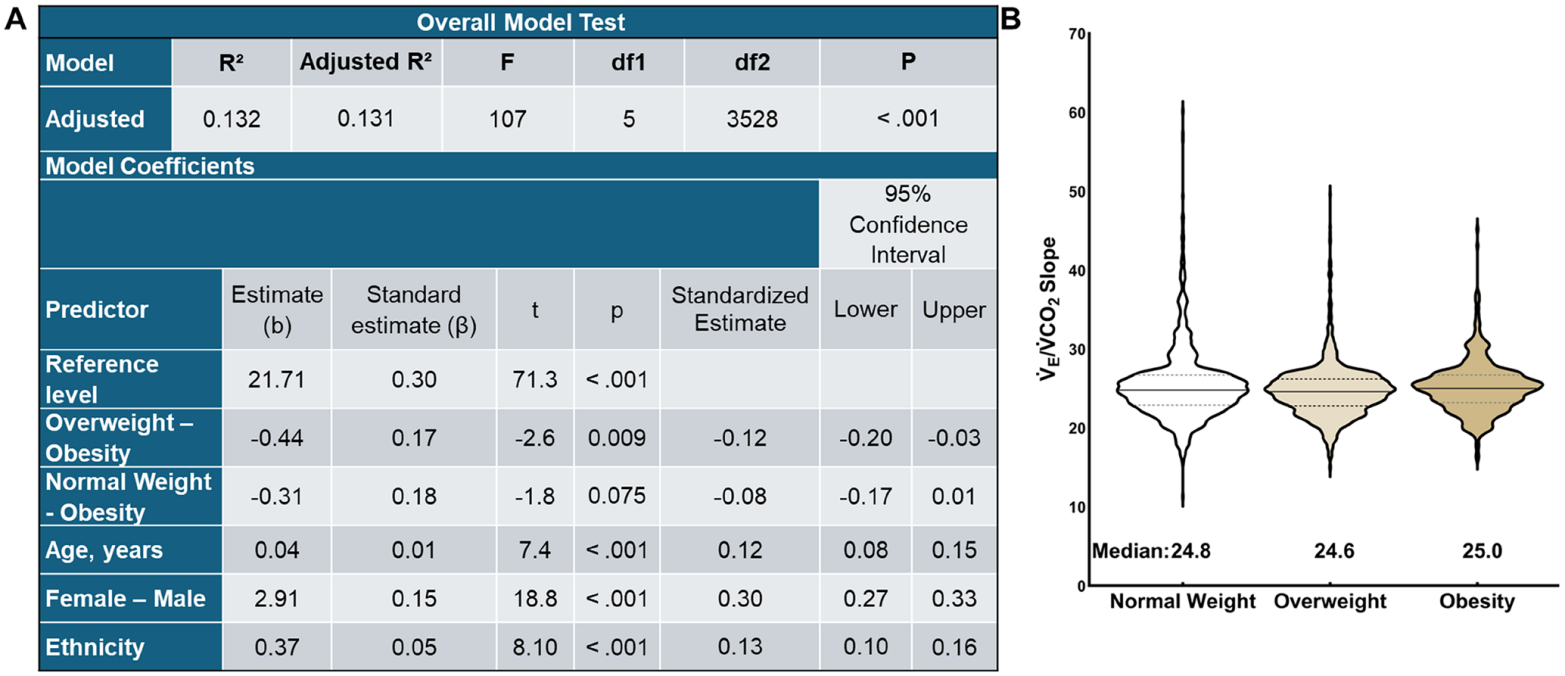
(A) Linear regression for V̇_E_/V̇CO_2_ slope in adults with normal weight, overweight, and obesity adjusted for age, sex, and race/ethnicity. (B) Violin plot of V̇_E_/V̇CO_2_ slope in adults without and with obesity. V̇_E_/V̇CO_2_ slope = minute ventilation/rate of carbon dioxide production.

In an unadjusted analysis, the V̇_E_/V̇CO_2_ slope was highest (*p* < 0.001) in adults with obesity and lowest in adults who were overweight (R^2^ = 0.006, adjusted R^2^ = 0.006, F_1,3532_ = 11.3, *p* < 0.001), but the effect size was small. Absolute V̇O_2peak_ was removed from the model because it was not a significant covariate (*p* = 0.807). In the covariate‐adjusted analysis, the V̇_E_/V̇CO_2_ slope differed between obesity subgroups compared to those without obesity (Figure 2), with a medium effect size. However, the difference between the highest and lowest V̇_E_/V̇CO_2_ slope was 0.4, which would not be a meaningful clinical difference.

Additionally, the proportion of adults with a V̇_E_/V̇CO_2_ slope > 45 did not differ between BMI subgroups (0.1%–0.5% prevalence per group, *X* ^2^ = 4.43, *p* = 0.109, Cramer's *V* = 0.035).

### 3.4 Association Between BMI and V̇_E_/V̇CO_2_ Slope

**FIGURE 3:**
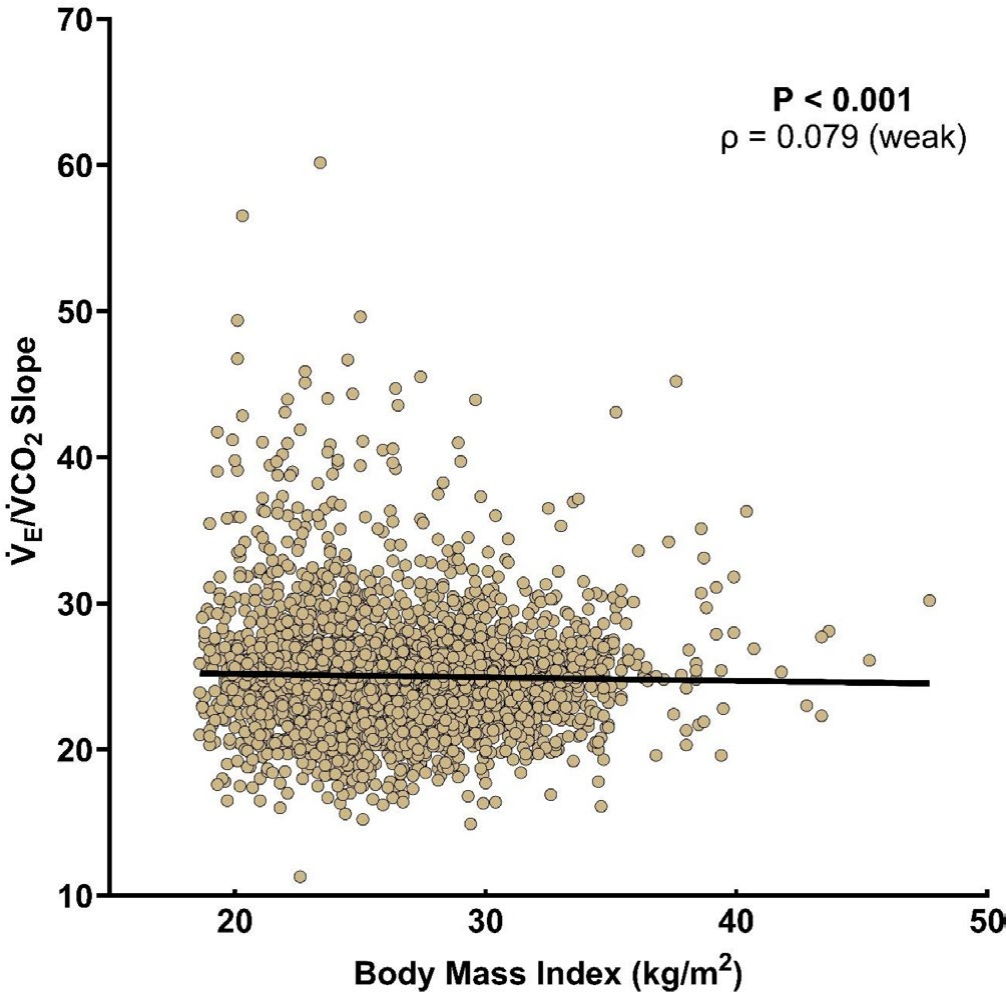
The correlation between body mass index and V̇_E_/V̇CO_2_ slope adjusted for age, sex, race/ethnicity, and absolute peak oxygen consumption rate. V̇_E_/V̇CO_2_ slope = minute ventilation/rate of carbon dioxide production.

In an unadjusted analysis, the V̇_E_/V̇CO_2_ slope was not associated with BMI (*p* = 0.857, ρ = 0.003). However, in the covariate‐adjusted analysis, the V̇_E_/V̇CO_2_ slope had a significant but weak correlation with BMI (Figure 3).

## 4 Discussion

Several important findings emerged from this analysis of > 3500 CPETs. First, adults with obesity had a greater V̇_E_/V̇CO_2_ slope compared to adults without obesity. Second, subgroup analyses suggested that adults who were overweight had lower V̇_E_/V̇CO_2_ slopes compared to adults with normal weight and obesity. However, these significant main findings all had negligible effect sizes, and the differences between groups are unlikely to signify a meaningful clinical difference, as the mean values are within the normal range. Third, when adjusted for age, sex, and ethnicity, there was a small positive correlation between BMI and the V̇_E_/V̇CO_2_ slope. Additionally, the prevalence of V̇_E_/V̇CO_2_ slopes > 45 did not differ between groups. Overall, these data suggest that BMI is not meaningfully associated with lower or higher ventilatory efficiency during exercise among apparently healthy adults.

Despite finding a significant relation between BMI and the V̇_E_/V̇CO_2_ slope when adjusted for relevant covariates, both the effect size and the clinical significance of the group differences were small. This suggests that in apparently healthy adults with obesity, BMI is only weakly associated with the V̇_E_/V̇CO_2_ slope. This differs from the findings of Balmain et al. [35], who found that the V̇_E_/V̇CO_2_ slope calculated up to the ventilatory threshold was lower in adults with obesity, with the degree of difference corresponding to the severity of obesity (30–80 kg/m^2^). The magnitude of obesity has been implicated as a major factor in the degree of respiratory irregularity, highlighting the heterogeneous impact of obesity on the work of breathing and ventilatory constraint [25, 53].

Our study leverages a large sample of adults with obesity from multiple geographical locations, but the highest BMI value in our sample (48 kg/m^2^) was lower than in previous studies [35, 54]. These divergent results highlight the need to examine the association between obesity and relevant health metrics at all BMI values. The present study examined apparently healthy adults with primarily stage 1 and 2 obesity, which is highly applicable as it constitutes the largest percentage of adults living with obesity in the United States (33%), as opposed to those living with stage 3 obesity (9%) [55]. The present study, by design, included adults with a lower‐than‐normal risk profile compared to average adults with obesity in order to control for comorbidities and to isolate BMI.

The fundamental cause of obesity is an energy intake and expenditure imbalance that results in excess adipose tissue manifesting in a myriad of physiological adaptations. Among these physiological adaptations are those that culminate in the development of chronic low‐grade systemic inflammation, a hallmark symptom of obesity [56]. In brief, the accumulation of visceral adipose tissue is accompanied by a transition of adipose phenotype, generally described as the shift from anti‐inflammatory to pro‐inflammatory [57]. In addition, recent studies have observed an association between obesity‐related inflammatory pathways and reduced lung function and exercise capacity [58]. The neutrophil‐to‐lymphocyte ratio (NLR) has been implicated as an inflammatory marker for adults with obesity [59, 60]. Moreover, NLR has been positively associated with increased mortality for the general population of the United States (~40% prevalence of obesity) [61]. Furthermore, emerging research has associated elevated NLR, as observed in obesity, with reduced CPET performance, as indicated by low V̇O_2peak_ and a high V̇_E_/V̇CO_2_ slope [62]. There is evidence to suggest that systemic inflammation impairs respiratory chemoreflexes [63], possibly impairing CO_2_ sensing during exercise. Interestingly, NLR appears to only be elevated in stage 3 obesity, with no changes in stage 1 and stage 2 compared to normal weight adults, further explaining the small effect size in our sample of adults, with mostly stage 1 and stage 2 obesity [64].

Adults with obesity exhibit reduced inspiratory muscle function and increased inspiratory muscle fatigue during exercise [65, 66, 67]. It has been proposed that a higher cost of breathing may in part explain the respiratory dysfunction observed in adults with obesity [65, 68]. This in part explains the high percentage of adults with obesity who experience dyspnea, with one epidemiological survey revealing that 80% of middle‐aged (37–57 years) adults with obesity experience dyspnea with activities of daily living (i.e., climbing stairs) [69]. Additionally, a high V̇_E_/V̇CO_2_ slope is associated with greater dyspnea [70]. However, dyspnea is complex and has numerous potential mechanisms. Adults with dyspnea on exertion, as indicated by CPET, were excluded from our analysis. However, we still observed reduced physical fitness in adults with obesity compared to those without. The effect of respiratory muscle dysfunction (e.g., inspiratory muscle weakness) in part explains the reduced cardiorespiratory fitness observed in adults with obesity [71]. Potentially, had our database included adults with dyspnea on exertion, our effect size may have been larger, given that greater dyspnea is associated with a high V̇_E_/V̇CO_2_ slope [70]. However, the several potential causes of dyspnea, along with more common comorbidities, would have confounded the interpretation of the primary research question addressed in this manuscript. Future studies are warranted to address this.

Recently, experts in the field of obesity research published comprehensive diagnostic criteria for pre‐clinical and clinical obesity [72]. This distinction differentiates individuals who exhibit an obese phenotype without any associated dysfunction from those whose obese phenotype is accompanied by dysfunction of tissue or organ systems. By design, our study focused on preclinical obesity because those with existing comorbidities were excluded. While our main findings are most generalizable to adults with a mild to moderate obesity phenotype free of chronic diseases, it is less generalizable to those with clinical obesity. Therefore, future studies should not only examine the full spectrum of obesity but also prioritize more precise categorization of participants using current clinical criteria to accurately identify individuals with clinical obesity.

### 4.1 Limitations

This was a retrospective study that leveraged the FRIEND database to isolate adults with and without obesity who were free of chronic diseases. Given the nature of this multicenter study spanning 30 years, it was not possible to standardize hardware, software, or the specific methods used for data collection. However, all studies followed the American College of Sports Medicine guidelines for conducting a CPET and are reliant upon well calibrated equipment for measuring gas concentrations and flow. We did not have access to comprehensive pulmonary function testing results. This limits the ability to determine ventilatory reserve volume or end‐expiratory lung volumes during peak exercise. Future studies should include pulmonary function testing to determine what level of ventilatory constraint is present at rest and during exercise. Additionally, our study lacked more comprehensive body composition metrics, including waist circumference, hip circumference, waist‐to‐hip ratio, and body fat percentage. Future studies should include more comprehensive body composition assessments. These additional metrics would provide details about body fat distribution and percentage to better elucidate if they are associated with V̇_E_/V̇CO_2_ slope.

### 4.2 Perspective

This study leverages a large sample (> 3500) of adults and found a significant positive correlation between BMI and V̇_E_/V̇CO_2_ slope. However, these findings were accompanied by small effect sizes, meaning that, despite revealing that adults with obesity had a higher V̇E/V̇CO2 slope, the magnitude of the difference was negligible and unlikely to be clinically meaningful. Our sample consisted of apparently healthy adults with and without obesity. Future work is needed to investigate the association between body composition (waist circumference, body fat distribution, etc.) and the V̇_E_/V̇CO_2_ slope. The nuances of calculating the V̇_E_/V̇CO_2_ slope from a CPET require future studies to present the slope across the entire test and up to submaximal cutoffs (e.g., ventilatory threshold).

## Author Contributions

Thomas G. Bissen and Joseph C. Watso conceived the research questions for this manuscript; all authors contributed to the acquisition, analysis, or interpretation of data; Thomas G. Bissen drafted the manuscript and all authors revised it critically for important intellectual content. All authors approved the final version of the manuscript. All authors agree to be accountable for all aspects of the work in ensuring that questions related to the accuracy or integrity of any part of the work are appropriately investigated and resolved. All persons designated as authors qualify for authorship, and all those who qualify for authorship are listed.

## Funding

Joseph C. Watso is supported by the NIH (K01HL160772) and the American Heart Association (23CDA1037938).

## Disclosure

Joseph C. Watso provides education/consulting at Watso Health LLC. All other authors have nothing to disclose.

## Conflicts of Interest

The results of the study are presented clearly, honestly, and without fabrication, falsification, or inappropriate data manipulation. The results of the present study do not constitute endorsement by the American College of Sports Medicine.

## Data Availability

The data that support the findings of this study are available on request from the corresponding author. The data are not publicly available due to privacy or ethical restrictions.
